# Opioid Use Disorder Significantly Increases Complications and Costs in Primary and Revision Total Knee Arthroplasty a Nationwide Analysis and the Case for Preoperative Screening

**DOI:** 10.3390/jcm14113832

**Published:** 2025-05-29

**Authors:** Ela Cohen Nissan, Yaara Berkovich, David Maman, Yaniv Yonai, Yaniv Steinfeld, Yaron Berkovich

**Affiliations:** 1 Faculty of Medicine, Technion Israel Institute of Technology, Haifa 2611001, Israel; ela.cohen@campus.technion.ac.il (E.C.N.); byaara@campus.technion.ac.il (Y.B.); yanivyonai@gmail.com (Y.Y.); yanivsteinfeld@gmail.com (Y.S.); yaron.berkovich@gmail.com (Y.B.); 2 Carmel Medical Center, Haifa 3436212, Israel

**Keywords:** total knee arthroplasty, opioid use disorder, Nationwide Inpatient Sample, surgical outcomes, complications

## Abstract

**Background:** Total knee arthroplasty (TKA) is one of the most frequently performed procedures for end-stage arthritis. Effective pain control is essential for recovery, and opioids are widely used. However, patients with opioid use disorder (OUD) may be at increased risk of complications. **Methods:** We analyzed 2,514,099 TKA cases from the Nationwide Inpatient Sample (2016–2019), identifying 11,785 patients with OUD. After 1:1 propensity score matching, clinical and economic outcomes were compared between OUD and non-OUD patients. **Results:** Patients with OUD had significantly higher odds of intraoperative fracture (OR: 6.1), DVT (OR: 5.0), pneumonia (OR: 2.5), pulmonary edema (OR: 1.6), and blood transfusion (OR: 1.5) (*p* < 0.001). Mean hospital charges were higher (USD 68,687 vs. USD 60,984), and LOS was longer (2.8 vs. 2.5 days, *p* < 0.001). OUD patients were more likely to undergo revision TKA at a younger age (59.6 vs. 65.4 years, *p* = 0.016), with higher infection rates and greater costs. **Conclusions:** Patients with OUD undergoing TKA experience more complications, higher costs, and require earlier revision. Underreporting of OUD highlights the need for improved preoperative screening.

## 1. Introduction

Total knee arthroplasty (TKA) stands out as one of the most effective surgical interventions for patients suffering with advanced arthritis. It is among the most frequently performed surgical procedures worldwide, demonstrating high patient satisfaction and a markedly rising incidence [1].

TKA is consistently associated with significant postoperative pain [2,3], which can impact early range of motion, patient satisfaction, and overall outcomes [2,3,4,5]. Opioids are effective and routinely prescribed as a standard component of pain management following TKA [2,3,6]. However, patients with OUD often exhibit heightened opioid tolerance, therefore, the perioperative management of these patients has become an increasing challenge [7,8,9].

Moreover, a diverse patient population is undergoing this surgery, including patients with different health conditions and functional needs. A portion of patients have a history of opioid use disorder (OUD), which introduces challenges in postoperative complications, hospital costs, hospital length of stay (LOS), and revision surgery rates [9,10,11]. However, physicians are not required to include questions about OUD in the preoperative questionnaire.

The opioid epidemic has become a severe public health crisis, causing significant social and economic impacts [12,13,14]. Between 2016 and 2019, the prevalence of OUD fluctuated, with an average reported rate of 1% of the United States population [15]. These rates continue to rise, highlighting the widespread impact of this disorder [12,13,15].

Previous research examining outcomes of TKA in patients with a history of OUD has been primarily based on small-scale studies, often with limited focus on postoperative complications. While the NIS is a publicly available resource, our study adds significant value by combining a modern dataset with a robust, large-scale matched analysis and an integrated economic model. Unlike prior studies that either lacked comprehensive matching or focused only on primary TKA, we uniquely assess both primary and revision TKA outcomes in patients with OUD, stratified by complication type, age at revision, and etiology. In addition, we provide a cost-effectiveness evaluation of preoperative OUD screening, which has not been addressed in the existing literature. This multifaceted approach, merging complication risk analysis, revision-specific trends, and modeled economic savings, provides a more clinically actionable framework for improving perioperative care for TKA patients with OUD.

TKA outcomes are comprehensively evaluated in patients with a history of OUD, and it is investigated whether they experience more postoperative complications, higher revision surgery rates, greater healthcare costs, and longer LOS compared to patients without OUD.

This study aims to evaluate the importance of preoperative screening for OUD and its potential impact on medical complications and healthcare costs. Identifying high-risk patients is crucial for predicting potential postoperative complications. Implementing targeted prevention strategies before and during hospitalization can significantly reduce complications and enhance patient recovery outcomes.

We hypothesize that patients with OUD undergoing TKA and revision surgery will experience more postoperative complications, higher healthcare costs, longer LOS, and higher revision surgery rates compared to those without OUD. Although preoperative screening for OUD is not currently mandatory, we think that implementing such screening could provide significant value.

### Research Question

Does a history of opioid use disorder independently predict worse clinical and economic outcomes in patients undergoing primary and revision total knee arthroplasty?

## 2. Methods

### 2.1. Dataset

We conducted our analysis using the Nationwide Inpatient Sample (NIS), a publicly accessible, all-payer database that represents the largest collection of inpatient hospital data in the United States. The primary analysis was conducted in 2024. The study population consisted of individuals who underwent either primary or revision total knee arthroplasty between 1 January 2016, and 31 December 2019. All analyses utilized the sampling weights (DISCWT) provided by the NIS to produce nationally representative estimates.

### 2.2. Patient Identification and Exclusions

Patients undergoing primary total knee arthroplasty and revision total knee arthroplasty were selected based on corresponding ICD-10 procedure codes. Opioid use disorder (OUD) was identified using the broad ICD-10 code F11.X. Specific subcategories (F11.1X, F11.2X, etc.) comprised a negligible proportion and were not analyzed separately. To ensure the analysis focused on elective surgeries, exclusions were applied to patients with non-elective admissions, those younger than 18 years old, and those undergoing revision procedures in the primary total knee arthroplasty cohort. A separate analysis was conducted for revision total knee arthroplasty cases.

### 2.3. Statistical Analyses and Propensity Score Matching

To control for confounding variables and ensure comparable groups, propensity score matching was performed. A one-to-one matching algorithm was applied to pair each patient with opioid use disorder with a patient without opioid use disorder, based on demographic data, comorbidities, and payer status. This resulted in two balanced cohorts, allowing for a direct comparison of outcomes.

All statistical analyses were conducted using SPSS version 26 and MATLAB 2024. Continuous variables were compared using independent *t*-tests, whereas categorical variables were analyzed with chi-square tests. A *p*-value of less than 0.05 was considered statistically significant. Length of stay (LOS), being skewed, represents a limitation as it was analyzed using standard parametric *t*-tests. Although this approach provides practical insights, future studies should utilize non-parametric methods or regression models such as negative binomial regression for improved accuracy.

### 2.4. Comorbidity and Outcome Identification

Comorbidities were identified using ICD-10 codes to assess differences between total knee arthroplasty patients with and without opioid use disorder. Key clinical outcomes included length of hospital stay, total hospitalization charges, and postoperative complications such as intraoperative fractures, venous thromboembolism, pneumonia, acute kidney injury, pulmonary edema, and blood transfusion requirements. To evaluate the association between opioid use disorder and complication risk, odds ratios along with 95% confidence intervals were computed.

### 2.5. Revision Surgery Analysis

A separate analysis was conducted for revision total knee arthroplasty patients. The revision TKA analysis was performed by the same research team. Among these cases, 1.4 percent of patients had opioid use disorder, compared to 0.5 percent in the primary total knee arthroplasty cohort (*p* < 0.001), suggesting an increased likelihood of revision among opioid users. Outcomes assessed included total hospital charges, length of stay, and the etiology of revision, with particular focus on infection rates and postoperative complications.

### 2.6. Cost Analysis of Universal Preoperative Urine Opioid Screening

To evaluate the cost-effectiveness of universal preoperative urine opioid screening, a cost analysis was performed. Additional hospital costs associated with opioid use disorder were compared to the estimated cost of a universal urine opioid test, set at USD 75 per patient. The financial impact was analyzed for both primary and revision total knee arthroplasty, assessing whether screening could reduce the economic burden of opioid-related complications.

### 2.7. Ethical Aspects

This study was exempt from institutional review board approval due to the use of de-identified data from the NIS database. Informed consent was not required, as the dataset does not contain patient-identifiable information.

## 3. Results

Table 1 presents the demographic and clinical characteristics of TKA patients with and without opioid use disorder. Patients with OUD were significantly younger (61.3 vs. 66.8 years, *p* < 0.001) and had a slightly lower proportion of female patients (60.6% vs. 61.6%, *p* = 0.034). They were more likely to have Medicaid as their primary payer and less likely to have private insurance (*p* < 0.001).

Comorbidities were more prevalent in the OUD group, with significantly higher rates of chronic lung disease, liver disease, and fibromyalgia (*p* < 0.001). Alcohol abuse was also notably more common in OUD patients.

### 3.1. A Propensity Score-Matched Evaluation of Total Knee Arthroplasty Outcomes in Patients with Versus Without Opioid Use Disorder

To minimize baseline imbalances and reduce selection bias, we employed a propensity score matching approach. This technique allowed for the comparison of TKA patients with and without opioid use disorder by aligning them on key demographic and clinical variables. Propensity score matching created statistically equivalent groups, balancing variables such as age, gender, and primary payer type, thereby minimizing confounding factors and improving the reliability of comparisons. After matching, no significant differences remained in age, gender distribution, or payer type, confirming the effectiveness of the matching process (Table 2). The prevalence of comorbidities, including hypertension, dyslipidemia, and diabetes, was also comparable between the groups, allowing for a more accurate comparison of postoperative outcomes.

### 3.2. Inpatient Outcomes Among Matched TKA Patients With and Without Opioid Use Disorder

After matching based on propensity scores, notable differences persisted in hospitalization metrics between the two cohorts (Table 3). Individuals with opioid use disorder experienced a longer mean length of stay (2.8 vs. 2.5 days, *p* < 0.001) and incurred greater total hospital charges (USD 68,687 vs. USD 60,984, *p* < 0.001).

### 3.3. Elevated Postoperative Complications in Opioid Use Disorder Patients Undergoing TKA

**Figure 1:**
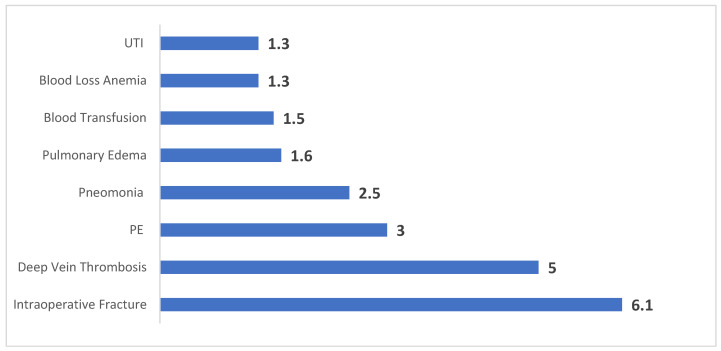
Odds of each postoperative complication following TKA in matched OUD vs. non-OUD patients.

Even after adjusting for baseline characteristics through propensity score matching, opioid use disorder remained strongly associated with elevated rates of several postoperative complications (Figure 1). Patients in the OUD group had more than a sixfold increased likelihood of sustaining intraoperative fractures (OR: 6.1, 95% CI: 4.0–9.3, *p* < 0.001) and a fivefold higher risk of developing deep vein thrombosis (OR: 5.0, 95% CI: 1.9–13.1, *p* < 0.001).

Pulmonary issues were also more prevalent among those with OUD. The incidence of pneumonia was 2.5 times greater (OR: 2.5, 95% CI: 1.2–5.2, *p* = 0.011), while pulmonary edema occurred 1.6 times more frequently (OR: 1.6, 95% CI: 1.4–1.8, *p* < 0.001). Furthermore, the OUD group showed significantly higher rates of blood transfusion (OR: 1.5, 95% CI: 1.3–1.8, *p* < 0.001) and blood loss anemia (OR: 1.3, 95% CI: 1.3–1.4, *p* < 0.001).

### 3.4. Revision TKA Outcomes in Patients With and Without Opioid Use Disorder

As shown in Table 4, among patients undergoing revision total knee arthroplasty, those with opioid use disorder accounted for 1.4% of all cases, compared to only 0.5% in primary TKA (*p* < 0.001), suggesting that opioid use disorder is associated with a significantly higher likelihood of requiring revision surgery.

OUD patients were also significantly younger at the time of revision, with an average age of 59.6 years compared to 65.4 years for non-OUD patients (*p* = 0.016), indicating a possible link between opioid use and earlier implant failure or complications necessitating revision.

Hospitalization outcomes demonstrated higher costs and longer hospital stays in the OUD group. Total charges were significantly elevated at USD 132,257 compared to USD 97,346 for non-OUD patients (*p* < 0.001), and the length of stay was prolonged at 4.4 days versus 3.1 days (*p* < 0.001). Age differences were addressed through logistic regression analysis to confirm that younger age independently increased revision risk, and this risk was significantly amplified by the presence of OUD. Interaction analyses highlighted significant contributions from chronic pulmonary disease, liver dysfunction, and renal dysfunction.

### 3.5. Primary Reasons for Revision TKA Among Patients With and Without Opioid Use Disorder

Table 5 highlights that infection was the most frequent indication for revision total knee arthroplasty in both cohorts, with a significantly higher prevalence among patients with opioid use disorder (32.3% vs. 22.1%, *p* < 0.001). In contrast, mechanical failure causes—such as loosening or prosthetic instability—were observed at slightly lower rates in the OUD group.

### 3.6. Postoperative Complications Following Revision TKA in Patients With and Without Opioid Use Disorder

**Figure 2:**
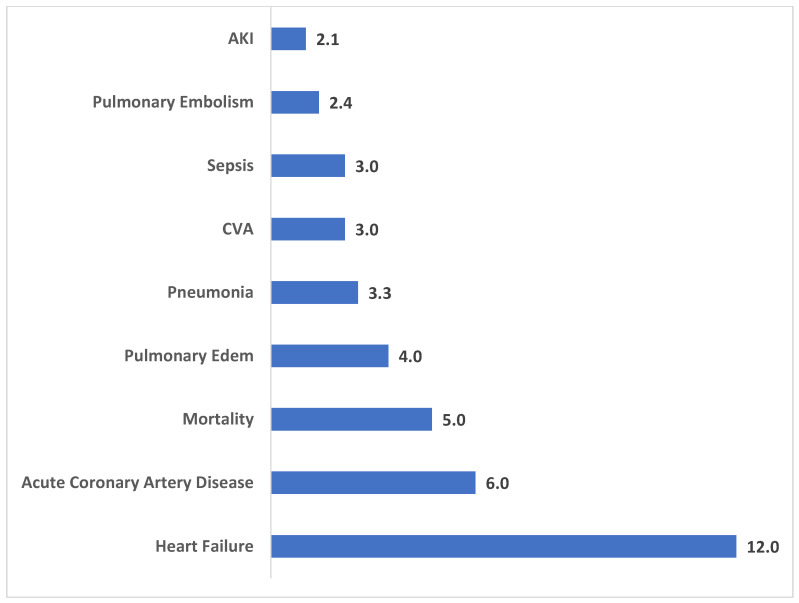
Odds of each postoperative complication following revision TKA OUD vs. non-OUD patients.

As shown in Figure 2, patients with opioid use disorder undergoing revision TKA experienced significantly higher rates of several postoperative complications. Pulmonary edema had the highest odds ratio (OR 3.7, 95% CI 2.0–7.1, *p* < 0.001), followed by deep vein thrombosis (OR 2.5, 95% CI 1.3–4.7, *p* = 0.003) and acute heart failure (OR 2.4, 95% CI 1.5–4.1, *p* < 0.001). Additionally, the risk of requiring blood transfusion was significantly elevated (OR 2.1, 95% CI 1.8–2.4, *p* < 0.001). Infectious complications also showed increased prevalence, with pneumonia (OR 1.9, 95% CI 1.0–3.5, *p* = 0.043) and revision due to infection (OR 1.7, 95% CI 1.6–1.8, *p* < 0.001) being notably higher in OUD patients.

### 3.7. Cost Impact of Opioid Use Disorder on TKA and the Potential Savings of Preoperative Screening

**Figure 3:**
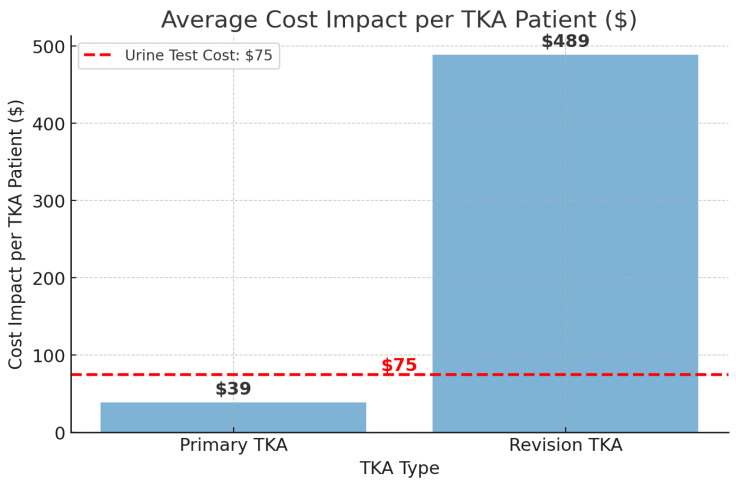
Cost impact of opioid use disorder on TKA and the potential savings of preoperative screening.

Figure 3 illustrates the average cost impact per TKA patient associated with opioid use disorder, reflecting the additional hospital costs incurred by this population. For primary TKA, the cost impact was USD 39 per patient, while for revision TKA, the additional cost reached USD 489 per patient, highlighting the substantial economic burden of opioid use disorder in orthopedic surgery. The left columns show potential savings from preoperative screening; right columns indicate added cost due to OUD-related complications.

The red dashed line represents the estimated cost of a universal preoperative urine opioid screening test at USD 75 per patient. This visual comparison suggests that, while the additional cost burden for primary TKA is lower than the screening cost, the significantly higher cost impact in revision TKA indicates that preoperative opioid screening could be a cost-effective strategy in this population.

## 4. Discussion

TKA is a widely performed surgical procedure for advanced arthritis. A subset of patients undergoing TKA has a history of opioid use disorder (OUD), a condition which can impact postoperative outcomes [9,10,11,16,17,18,19,20]. We analyzed TKA outcomes in patients with OUD using big data to assess complications, costs, and hospital stays compared to individuals without OUD.

### 4.1. Key Findings and Clinical Implications

Our data reveals significant underreporting of OUD in the preoperative setting. The most important findings of this study were that patients with OUD undergoing TKA experience significantly higher rates of postoperative complications, including intraoperative fractures, deep vein thrombosis, pulmonary embolism, and pneumonia, as well as increased total hospital charges and LOS compared to patients without OUD. Furthermore, revision TKA surgeries were more prevalent among patients with OUD and occurred at a younger average age compared to those without OUD. OUD patients had a significantly higher risk of infection as the primary etiology for revision, longer LOS, and higher hospitalization costs. These findings underscore the importance of accurate preoperative identification and individualized perioperative management of OUD to improve outcomes and reduce costs in TKA care.

Orthopedic teams should be aware of the underreporting of OUD and prioritize early identification as a key step toward optimizing surgical care. Individualized perioperative protocols are recommended. Including bone health assessment, personalized pain management strategies, and modifications in surgical and pharmacological approach [3,5,6,8].

### 4.2. Under-Reporting of OUD in Preoperative Screening

Our analysis of data collected from the NIS between 2016 and 2019 indicates that the prevalence of OUD among TKA patients was 0.5% (Figure 1). However, during the same period, the estimated average prevalence of OUD in the general United States population was 1% [15]. This underscores the insufficient inquiry by physicians regarding OUD, resulting in an incomplete understanding of the patient’s medical history prior to surgery, which may negatively impact both the surgical procedure and its outcomes. These findings align with the lack of a mandatory requirement for inquiring about OUD [9,10].

### 4.3. Increased Postoperative Complications and Healthcare Costs

Our research reveals an elevated risk of complications such as intraoperative fractures, deep vein thrombosis, pulmonary embolism, and pneumonia. These results are consistent with previous studies, highlighting that patients with OUD face a notably higher risk for these complications [9,10,11,20].

Total hospital charges were significantly higher for the patients with OUD compared to the patients without OUD (USD 68,687 vs. USD 60,984, *p* < 0.001). Moreover, a notable disparity was observed in the LOS between patients in the OUD group compared to the non-OUD group (2.8 vs. 2.5 days, *p* < 0.001). These results are consistent with previous research [10,16,20]. Complications may arise due to direct opioid effects on bone metabolism (opioid endocrinopathy) increasing fracture risk, and through exacerbated postoperative recovery challenges due to prevalent comorbidities such as chronic pulmonary and liver diseases [20,21,22].

This implies that patients undergoing TKA with OUD place an increased economic burden on the healthcare system, emphasizing the importance of accurately identifying all patients with OUD and mitigating the prevalence of underreporting. Confronting this issue is crucial for optimizing healthcare efficiency and improving clinical decision-making [23].

### 4.4. Increased Risk of Intraoperative Fracture

The odds of intraoperative fracture were over six times higher in patients with OUD undergoing TKA than in patients without OUD (OR: 6.1, 95% CI: 4.0–9.3, *p* < 0.001). Those findings can be attributed to opioid endocrinopathy, a condition that disrupts bone metabolism and contributes to the development of osteoporosis [21,22]. Moreover, as documented in the literature, patients with osteoporosis face an increased risk of intraoperative fractures during TKA [21,22]. These findings underscore the importance of identifying OUD preoperatively, which can influence pharmacological treatment options or necessitate modifications in the surgical approach.

### 4.5. Reoperations and Extended Postoperative Outcomes

OUD is associated with a higher likelihood of undergoing revision surgery. (1.4% of all cases vs. 0.5% in primary TKA (*p* < 0.001). Moreover, patients with OUD were also significantly younger at the time of revision (59.6 years vs. 65.4 years (*p* = 0.016)), indicating a possible link between opioid use and earlier implant failure or complications necessitating revision. These results are consistent with previous studies 23,24]. Furthermore, revision surgeries in patients with OUD demonstrated higher costs in hospitalization (USD 132,257 vs. USD 97,346 (*p* < 0.001)) and longer LOS (4.4 days vs. 3.1 days (*p* < 0.001)).

In our study, infection was the primary etiology differentiating patients with OUD from those without (32.3% to 22.1%, respectively). The association between preoperative opioid use and an increased risk of infection has been documented [9,10,20]. This correlation is likely driven by a combination of patient-related factors and socioeconomic influences. While the underlying mechanisms are not yet fully understood, existing evidence indicates that opioid use may hinder the wound healing process [9,10,15,16,22]. Additional research is required to further investigate the correlation.

### 4.6. Limitations

This study has several limitations. The NIS database provides extensive healthcare data, but it may contain errors due to suboptimal coding, underreporting, incomplete documentation, and manual entry. Additionally, the NIS only provides data for the in-hospital period, limiting the ability to assess long-term outcomes. Future studies should include long-term follow-up to better understand the full impact of OUD history on TKA Surgery outcomes.

Another limitation is the lack of data regarding the dosage of opioid and duration of OUD within patients. Opioid endocrinopathy and wound healing processes are influenced by the intensity and chronicity of opioid use [11,21,22,24]. OUD includes a wide range of use patterns, therefore incorporating these factors might have influenced the outcomes. We acknowledge limitations regarding the lack of detailed severity distinctions within OUD subcategories (F11.X codes), potentially influencing outcomes and analysis granularity.

Despite these limitations, the strengths of this study include the use of a large, nationally representative dataset and the application of rigorous statistical methods to ensure robust and reliable results. The findings underscore the importance of early identification of individuals with OUD, both to enhance clinical outcomes and to mitigate the associated burden on healthcare systems.

## 5. Conclusions

Our study demonstrates that patients with a history of OUD experience worse postoperative outcomes, including a significantly higher risk of complications such as intraoperative fractures, thromboembolic events, pneumonia, and infections [25]. These patients also incur substantially greater hospital charges, extended lengths of stay, and elevated revision surgery rates, often at a younger age and with higher associated costs and LOS. The pronounced underreporting of OUD in preoperative assessments emphasizes the need for improved screening protocols to ensure accurate patient evaluation. Surgeons should implement screening protocols for OUD to enable early identification, thereby improving surgical outcomes. Future studies should further explore the long-term impact of OUD on TKA outcomes, with particular focus on the role of opioid endocrinopathy, preoperative optimization strategies, and the management of opioid use in surgical candidates to reduce the preoperative risk of complications and enhance overall patient outcomes.

## Figures and Tables

**Table 1:** Demographic, payer characteristics, and comorbidity comparison in TKA patients with and without opioid use disorder.

Clinical Variable	No Opioid Use Disorder	Opioid Use Disorder	*p*-Value
Total Procedures	2,502,314 (99.5%)	11,785 (0.5%)	-
Mean Patient Age (years)	66.8	61.3	<0.001
Female Sex (%)	61.6	60.6	0.034
Insurance Type—Medicare (%)	57.1	54.4	<0.001
Insurance Type—Medicaid (%)	4.2	15.1	
Insurance Type—Private/HMO (%)	35.1	26.1	
Insurance Type—Self-pay (%)	0.5	0.3	
Insurance Type—No Charge (%)	0	0	
Insurance Type—Other (%)	3.1	4.0	
Hypertensive Disease (%)	59.6	57.1	<0.001
Lipid Metabolism Disorders (%)	46.7	38.5	<0.001
Sleep Apnea Syndrome (%)	13.2	16.5	<0.001
Chronic Blood Loss Anemia (%)	5.8	7.7	<0.001
Alcohol Use Disorder (%)	0.9	4.8	<0.001
Fragility Fracture Risk (Osteoporosis) (%)	4.0	4.5	<0.001
Parkinsonian Syndromes (%)	0.6	0.6	0.825
Neurocognitive Disorders (%)	0.2	0.2	0.978
Renal Dysfunction (%)	6.9	10.0	<0.001
Congestive Cardiac Failure (%)	1.2	2.0	<0.001
Chronic Pulmonary Disease (%)	5.9	14.7	<0.001
Prior Myocardial Infarction (%)	3.1	3.8	<0.001
Diabetes (any type) (%)	21.7	22.5	0.022
Hepatic Dysfunction (%)	1.2	4.3	<0.001
Fibromyalgia Diagnosis (%)	2.7	11.2	<0.001
Endocrine—Thyroid Disorders (%)	17.9	18.4	0.825

**Table 2:** Propensity score-matched comparison of TKA patients with and without opioid use disorder.

Clinical Variable	No Opioid Use Disorder	Opioid Use Disorder	*p*-Value
Total Procedures	11,785	11,785	-
Mean Patient Age (years)	61.3	61.3	*p* = 0.59
Female Sex (%)	61.1	60.6	*p* = 0.51
Insurance Type—Medicare (%)	54.8	54.4	*p* = 0.91
Insurance Type—Medicaid (%)	14.7	15.1	
Insurance Type—Private/HMO (%)	26.3	26.1	
Insurance Type—Self-pay (%)	0.3	0.3	
Insurance Type—No Charge (%)	0	0	
Insurance Type—Other (%)	3.9	4	
Hypertensive Disease (%)	57.7	57.1	*p* = 0.36
Lipid Metabolism Disorders (%)	37.8	38.5	*p* = 0.28
Sleep Apnea Syndrome (%)	16.5	16.5	*p* = 0.93
Chronic Blood Loss Anemia (%)	7.7	7.7	*p* = 0.99
Alcohol Use Disorder (%)	4	4.8	*p* = 0.20
Fragility Fracture Risk (Osteoporosis) (%)	4.5	4.5	*p* = 1
Parkinsonian Syndromes (%)	0.6	0.6	*p* = 0.86
Neurocognitive Disorders (%)	0.2	0.2	*p* = 1
Renal Dysfunction (%)	9.6	10	*p* = 0.33
Congestive Cardiac Failure (%)	1.8	2	*p* = 0.46
Chronic Pulmonary Disease (%)	13.9	14.7	*p* = 0.08
Prior Myocardial Infarction (%)	3.4	3.8	*p* = 0.12
Diabetes (any type) (%)	23.2	22.5	*p* = 0.16
Hepatic Dysfunction (%)	3.8	4.3	*p* = 0.38
Fibromyalgia Diagnosis (%)	10.4	11.2	*p* = 0.10
Endocrine—Thyroid Disorders (%)	17.9	18.4	*p* = 0.07

**Table 3:** Hospitalization outcomes in propensity score-matched TKA patients with and without opioid use disorder.

	No Opioid Use Disorder	Opioid Use Disorder	*p* Value
Mean LOS in days	2.5 (S.D 1.0)	2.8 (S.D 3.0)	*p* < 0.001
mean charges in $	60,984 (S.D 36,232)	68,687 (S.D 41472)	*p* < 0.001

**Table 4:** Revision TKA outcomes in patients with and without opioid use disorder.

Revision Surgery	No Opioid Use Disorder	Opioid Use Disorder	Significance
Total numbers	220,215 (98.6%)	3030 (1.4%)	
Age at Revision (Years)	65.4 (Std Deviation 10.4)	59.6 (Std Deviation 10.2)	*p* = 0.016
Total charges (USD)	97,346 (Std Deviation 76,718)	132,257 (Std Deviation 413,396)	*p* < 0.001
Length of stay (Days)	3.1 (Std Deviation 2.8)	4.4 (Std Deviation 5.2)	*p* < 0.001

**Table 5:** Etiology of revision in patients with and without opioid use disorder.

Etiology for Revision	No Opioid Use Disorder	Opioid Use Disorder	
Infection	22.1%	32.3%	*p* < 0.001
Mechanical Loosening	23.0%	19.0%	
Pain	7.4%	5.6%	
Instability of Prosthesis	12.1%	10.1%	
Wear of Articular Surface	2.1%	1.5%	
Periprosthetic Fracture	0.9%	0.2%	
Fibrosis due to Prosthetic	0.9%	0.7%	
Broken Prosthesis	1.1%	0.7%	
Wound complication	0.4%	0.3%	
Mechanical Complication	9.5%	8.9%	
Other/Unspecified	20.6%	20.8%	

## Data Availability

The original contributions presented in this study are included in this article, further inquiries can be directed to the corresponding author.

## Abbreviations

AKI	Acute Kidney Injury
DVT	Deep Vein Thrombosis
HMO	Health Maintenance Organization
ICD-10	International Classification of Diseases, 10th Revision
LOS	Length of Stay
NIS	Nationwide Inpatient Sample
OUD	opioid use disorder
PE	Pulmonary Embolism
TKA	Total Knee Arthroplasty
